# The level of netrin-1 is decreased in newly diagnosed type 2 diabetes mellitus patients

**DOI:** 10.1186/s12902-016-0112-z

**Published:** 2016-06-02

**Authors:** Chenxiao Liu, Xianjin Ke, Ying Wang, Xiu Feng, Qi Li, Ying Zhang, Jian Zhu, Qian Li

**Affiliations:** Department of Endocrinology, Nanjing First Hospital, Nanjing Medical University, 68 Changle Road, Qinhuai District, Nanjing, Jiangsu Province 210006 China; Department of Neurology, Affiliated Hospital of Jiangsu University, Zhenjiang, China

**Keywords:** Netrin-1, Mechanism, Insulin resistance, Inflammation, Type 2 diabetes mellitus

## Abstract

**Background:**

Pancreatic β-cell dysfunction resulting from inflammation has been recognized to contribute to type 2 diabetes mellitus (T2DM). Netrin-1 is a new indicator of subclinical inflammation and it has a role in β-cell apoptosis. This study evaluated the level of netrin-1 in newly diagnosed T2DM patients and explored whether netrin-1 is a reliable marker or a key factor in the development of T2DM.

**Methods:**

Netrin-1 level was determined using a commercially available human enzyme-linked immune sorbent assay (ELISA) kit. The homeostasis model assessment of insulin resistance (HOMA-IR) was used as an index to measure insulin resistance. The sample consisted of 30 patients with newly diagnosed T2DM who had a glycosylated hemoglobin (HbA_1c_) level ranging from 7.5 % (58 mmol/mol) to 10.5 % (91 mmol/mol). The control group consisted of 26 healthy individuals matched for age and body mass index.

**Results:**

The netrin-1 level of T2DM patients was significantly lower than that of healthy controls (*p* < 0.01). Logistic regression analysis showed that the level of netrin-1 was negatively correlated with HOMA-IR, fasting blood glucose, postprandial blood glucose, fasting insulin and HbA_1c_.

**Conclusions:**

Plasma netrin-1 levels were decreased in patients with newly diagnosed T2DM, and the levels of netrin-1 were negatively associated with IR and glucose homeostasis. Future studies on the precise mechanism will offer new insights into the prevention and treatment of T2DM.

## Background

Insulin resistance (IR) and pancreatic β-cell dysfunction lead to type 2 diabetes mellitus (T2DM) [1–3]. IR leads to the inability of insulin to act normally in regulating nutrient metabolism in peripheral tissues. In recent years, increasing evidence from human population studies and animal research has pointed to a correlative and causative relationship between inflammation and IR/T2DM [4–6]. IR might promote inflammation by impairing the anti-inflammatory effect of insulin. Meanwhile, cytokines in turn enhance IR in adipose and other tissues, increasing the risk of T2DM [7]. Netrin-1, a neuroimmune guidance cue, plays a major role in the development of embryonic pancreas [8–10]. It associates with integral proteins α6β4 and α3β1 and inhibits pancreatic epithelial cell adhesion and migration [11], which differs from its function in neuronal cell migration. In addition to inhibition of migration, netrin-1 also suppresses inflammatory cytokine and chemokine production [9]. Recently, some studies have suggested that netrin-1 suppresses infiltration and inflammation in sepsis, acute kidney injury, acute lung injury and peritoneal inflammation [12, 13]. Other studies indicate that netrin-1 might regulate cyclooxygenase-2 (COX-2) expression through inhibition of nuclear factor kappa B activation [14] and promote macrophage differentiation to the M2-like phenotype [15].

Therefore, netrin-1 may be involved in the inflammatory mechanism of IR. In this article, we measured plasma netrin-1 levels in newly diagnosed patients with T2DM.

### Study population and design

Conducted in the Department of Endocrinology of Nanjing First Hospital, Nanjing Medical University, China from January to June 2013, our study included 30 patients newly diagnosed with T2DM. All of the patients had been diagnosed with T2DM within 6 months and had not received previous anti-hyperglycemic therapy. Diagnosis of T2DM was based on World Health Organization diagnostic criteria from 1999 [16]. Exclusions included: (1) patients prescribed any oral hypoglycemic agents or insulin; (2) impaired glucose tolerance or impaired fasting glucose; (3) a history of acute diabetic complications (such as diabetic ketoacidosis, hyperglycemic hyperosmolar status and diabetic lactic acidosis); (4) severe uncontrolled hypertension (systolic blood pressure ≥180 mmHg and/or diastolic blood pressure ≥110 mmHg); (5) any acute inflammation or infection; (6) current or a history of significant co-morbid diseases, such as cardiovascular (including myocardial infarction, cardiac surgery or revascularization, angina and congestive heart failure), hepatic and renal conditions; and (7) positivity for islet cell autoantibodies (such as glutamic acid decarboxylase autoantibody, islet cell autoantibody or insulinoma-like antigen 2) that indicate the possibility of type 1 diabetes mellitus. The control group comprised 26 aged-matched healthy subjects (all subjects with normal glucose tolerance at the baseline examination). All participants were surveyed on the following: age, sex, height, weight, smoking, drugs used, hyperlipidemia, and dietary compliance.

The study was approved by the appropriate independent ethics committees and regulatory authorities, and was conducted in accordance with the Declaration of Helsinki [17] and Good Clinical Practice guidelines [18]. All subjects provided informed consent prior to being enrolled and after the purpose and procedures of the study were fully explained. Written informed consent was obtained from all patients.

### Methods

Baseline clinical and demographic data for all of the study participants were collected from the medical records. Body mass index (BMI) was calculated as weight (kg)/square of height. Venous blood samples were obtained from all of the participants after 8-h fasting and treated separately. Postprandial blood glucose (PBG) and postprandial insulin concentrations (PINS) were assessed 2 h after a test meal. Hematology and biochemistry were determined by routine techniques using an automated analyzer. Blood was collected in 4 mL EDTA containers and was centrifuged within 30 min at 10000 rpm for 10 min. Serum samples were subsequently stored in aliquots without preservatives at −80 °C for an average of 3 months until immediately before analysis of netrin-1. Netrin-1 level was determined using a commercially available human enzyme-linked immune sorbent assay (ELISA) kit, purchased from Wuhan Huamei Biological Engineering Co., Ltd.

Plasma glucose levels, sodium concentrations, insulin levels, blood lipid profiles and glycosylated hemoglobin (HbA_1c_) levels were assayed by routine methods. The homeostasis model of IR (HOMA-IR) was used as a measure of IR. HOMA-IR was calculated using the following formula: fasting plasma glucose (mmol/L) multiplied by fasting serum insulin (mIU/L) divided by 22.5.

### Definitions

Diabetes was diagnosed based on the World Health Organization consulting criteria [16] (i.e., fasting plasma glucose [FPG] of ≥7.0 mmol/L [126 mg/dL] and/or a 2-h post glucose value of ≥11.1 mmol/L [200 mg/dL]).

### Statistical analysis

Data were analyzed with the SPSS 20.0 program. For continuous variables with normal distributions, data were expressed as mean ± standard deviation and non-normally distributed variables (FBG, PBG, HbA_1c,_ low-density lipid [LDL] cholesterol) were expressed as median (interquartile range). The Kolmogorov–Smirnov test was used to evaluate the distribution of variables. Student’s *t*-test (independent-sample *t*-test) was used for continuous variables with normal distribution. Non-normally distributed variables have been log-transformed. Pearson’s correlation analyses were used to assess the relationships. Logistic regression analysis was used to assess the associations between netrin-1 level and the other parameters evaluated. A value of *p* < 0.05 was accepted as the level of significance (two-tailed).

## Results

The groups were similar in terms of age, sex, smoking history, drugs used and BMI. The T2DM group showed significantly higher FBG, PBG, FINS and HbA_1c_ values than the control group. No significant differences in triglyceride (TG), total cholesterol (TC), high-density lipid (HDL) cholesterol, and LDL cholesterol levels were detected between the two groups. The demographic and laboratory data of the groups is outlined in Table 1.

**Table 1:** Demographic and laboratory data of the patient and control groups

Variables	T2DM (*n* = 30)	Control (*n* = 26)	*P* value
Gender (M/F)	16/14	13/13	
Age (years)	52.7 (11.08)	52.96 (11.65)	0.932
Height (cm)	1.69 (0.89)	1.64 (0.87)	0.057
Weight (Kg)	66.13 (6.03)	65.92 (9.16)	0.920
PBG (mmol/L)	13.35 (11.40,15.23)	6.85 (6.10,7.20)	0.000
BMI (Kg/m^2^)	23.19 (2.41)	24.21 (2.58)	0.122
FBG (mmol/L)	8.50 (7.70,9.00)	5.14 (4.65,5.42)	0.000
HbA_1c_ (%)	8.50 (7.90,9.10)	5.40 (5.30,5.85)	0.000
HDL-C (mmol/L)	1.22 (0.36)	1.20 (0.33)	0.860
LDL-C (mmol/L)	2.82 (2.04,3.73)	2.81 (1.84,3.73)	0.792
TC (mmol/L)	4.90 (0.79)	4.92 (0.88)	0.932
TG (mmol/L)	1.89 (1.05)	1.50 (0.36)	0.084
FINS (pmol/L)	20.39 (6.36)	16.72 (6.04)	0.032
PINS (pmol/L)	57.06 (2.48)	114.58 (32.58)	0.000
HOMA-IR	1.13 (0.39)	0.54 (0.20)	0.000
Netrin-1 (pg/mL)	0.96 ± 0.5	1.77 ± 1.18	0.001

All parameters were expressed as mean ± SD (minimum–maximum) values unless otherwise stated. *P* < 0.05 was accepted as the level of significance

*FBG* Fasting Blood Glucose, *PBG* Postprandial Blood Glucose, *HbA*_*1c*_ glycosylated hemoglobin, *TG* triglyceride, *TC* Total Cholesterol, *HDL-C* high density lipid-cholesterol, *LDL-C* low density lipid-cholesterol, *FINS* fasting insulin, *PINS* postprandial insulin, *HOMA*-*IR* homeostasis model assessment of insulin resistance

**Fig. 1:**
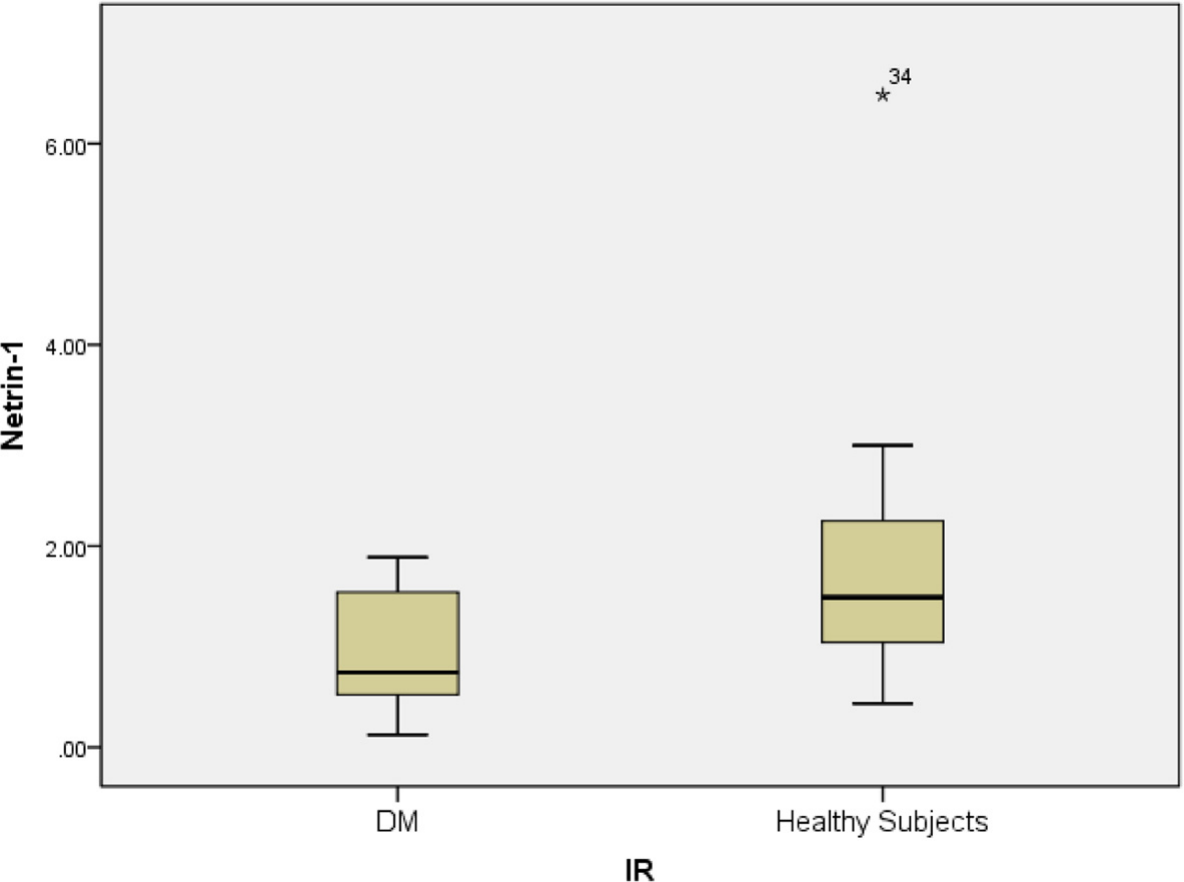
Mean netrin-1 values of the groups

**Fig. 2:**
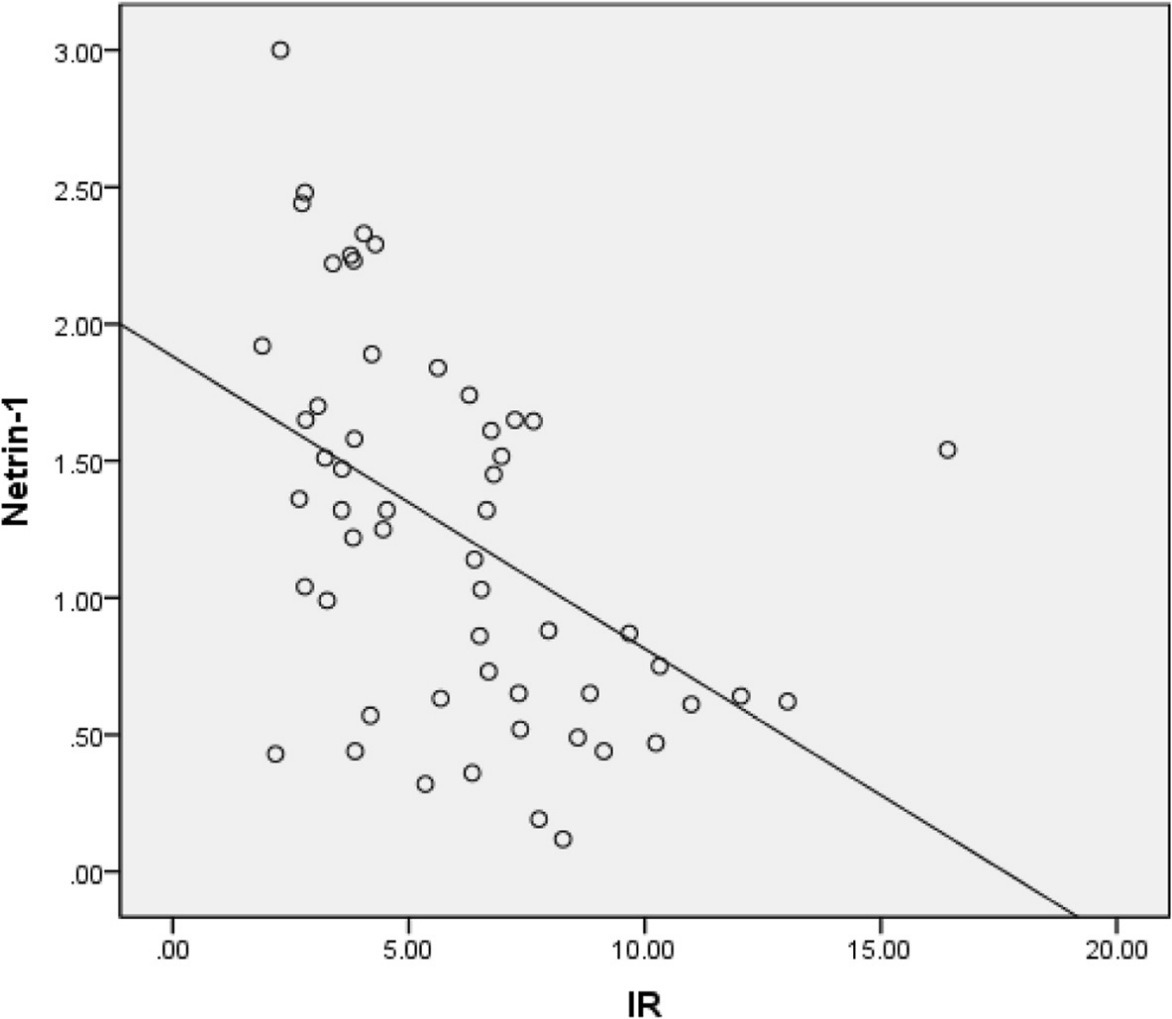
The association between netrin-1 and insulin resistance (IR)

The netrin-1 levels of the T2DM group were significantly lower than those of the control group (Table 1, Fig. 1). Netrin-1 levels showed significant negative correlation with HOMA-IR (*r* = −0.413, *p* < 0.001, Fig. 2).

A logistic regression analysis was also carried out using the enter method to evaluate the impact of netrin-1 on modulation of glucose homeostasis. The measurement of TG (*r* = −0.248, *p* = 0.032), FBG (*r* = −0.408, *p* = 0.001), PBG (*r* = −0.299, *p* = 0. 013), HbA_1c_ (*r* = −0.346, *p* = 0.005), FINS (*r* = −0.293, *p* = 0.014) and PINS (*r* = 0.357, *p* = 0.003) were dependent parameters, whereas age, sex, BMI, TC, HDL -C and LDL -C were independent parameters. The results showed that netrin-1 was independently related to IR and glucose homeostasis. Data are shown in Table 2.

**Table 2:** Logistic regression analysis of factors associated with netrin-1

Variable	*r*	*P* value
FBG	−0.408	0.001
PBG	−0.299	0.013
HbA_1C_	−0.346	0.005
HDL-C	0.145	0.144
LDL-C	−0.018	0.449
TG	−0.248	0.032
TC	−0.028	0.418
BMI	0.146	0.142
FINS	−0.293	0.014
PINS	0.357	0.003
HOMA-IR	−0.413	0.001

*FBG* Fasting Blood Glucose, *PBG* Postprandial Blood Glucose, *HbA*_*1c*_ glycosylated hemoglobin, *TG* triglyceride, *TC* Total Cholesterol, *HDL-C* high density lipid-cholesterol, *LDL-C* low density lipid-cholesterol, *FINS* fasting insulin, *PINS* postprandial insulin, *HOMA*-*IR* homeostasis model assessment of insulin resistance

*P* < 0.05 was accepted as the level of significance

## Discussion

The present study showed that the level of netrin-1 in patients with T2DM was significantly lower than that of the control group (*p* < 0.001). In addition, the level of netrin-1 was negatively related with FBG, PBG, HbA_1c_, FINS, TG and HOMA-IR.

Netrin-1 level was reduced and negatively correlated with blood glucose levels, IR and TG in T2DM patients. There may be three possible explanations for this finding. First, netrin-1 has a role in inflammation, which is implicated in the pathogenic mechanism of T2DM. Natura r [14] showed that netrin-1 could regulate inflammation, which might negatively regulate insulin secretion and contribute to β-cell dysfunction. Ranganathan et al. [19] also reported that netrin-1 regulates COX-2 expression at the transcriptional level. At the same time, overexpression of netrin-1 promotes macrophage differentiation to the “alternative” or M2-like phenotype and promotes islet remodeling [15]. Second, netrin-1 is also associated with islet dysfunction in diabetes and negatively correlated with hyperglycemia. It is understandable that repeated and prolonged exposure to hyperglycemia leads to β-cell degradation, reduces glucose-stimulated insulin secretion and eventually causes β-cell apoptosis [20]. De Breuck et al. [15] had suggested that netrin-1 is expressed and secreted in the pancreas where it plays a major role in pancreatic morphogenesis in the regenerating pancreas. In patients with newly diagnosed T2DM, secretion of netrin-1 in the injured and apoptotic β cells is significantly reduced, in turn promoting β-cell function failure. Third, IR disrupts the circulation of netrin-1. Ramkhelawon et al. [21] have suggested that netrin-1 is only selectively modestly upregulated in the visceral white adipose tissue, and that significant reductions in circulating levels of netrin-1 occur in obese individuals compared with lean individuals. They also studied obese rats and showed that netrin-1 is a macrophage retention signal in adipose tissue during obesity, which possibly promotes the chronic inflammation and insulin resistance that subsequently occurs in T2DM.

Data from this study suggest that netrin-1 is negatively correlated with blood glucose levels and IR. In addition, De Breuck et al. [15] also suggested that netrin-1 increases pancreatic islet cell mass and density in T2DM, which showed that netrin-1 possibly has a protective role in β-cell to delay the progression of this disease. If supplied with exogenous netrin-1 to patients could increase insulin sensitivity and improve insulin resistance. In the event of this regard, netrin-1 may be useful in treating patients with T2DM or without diabetes but with insulin resistance.

Our study has some limitations that should be considered in the interpretation of these results. First, the sample size is relatively modest. Second, we detected netrin-1 levels only in humans but not in animals. The receptors of netrin-1 have been widely shown to affect the inflammatory response, and changes in the expression levels of netrin-1 receptors could have confounded the results. Lastly, HOMA-IR is difficult to elucidate, causal relationships cannot be identified in cross-sectional studies, and there are no comparative patient groups, such as pre-diabetes. Despite these limitations, the safety and feasibility results of this study support further investigations to evaluate the influence of netrin-1 in the pathogenesis of T2DM.

## Conclusions

This study provides new evidence that netrin-1 participates in the development of T2DM and facilitates future studies to focus on the precise mechanism. Further study of netrin-1 could offer new insights into the prevention and treatment of T2DM.

## Abbreviations

FBG, fasting blood glucose; PBG, postprandial blood glucose; HbA_1c_, glycosylated hemoglobin; TG, triglyceride; TC, total cholesterol; HDL-C cholesterol, high-density lipid cholesterol; LDL-C cholesterol, low-density lipid cholesterol; FINS, fasting insulin; PINS, postprandial insulin; HOMA-IR, homeostasis model assessment of insulin resistance; BMI, body mass index; IR, insulin resistance; T2DM, type 2 diabetes mellitus; COX-2, cyclooxygenase-2
